# Illinois

**Published:** 1896-05

**Authors:** 


*Illinois.* In a herd of thirty-eight, tested by State Veterinarian Trumbower, four responded, were killed, and found to be extensively affected with tuberculosis.

